# PD-1 blockade combined with gemcitabine plus nab-paclitaxel is superior to chemotherapy alone in the management of unresectable stage III/IV pancreatic cancer: a retrospective real-world study

**DOI:** 10.3389/fonc.2023.1281545

**Published:** 2023-10-27

**Authors:** Daoan Cheng, Jing Hu, Xiaoyu Wu, Banglu Wang, Rui Chen, Weiqing Zhao, Cheng Fang, Mei Ji

**Affiliations:** Departments of Oncology, The Third Affiliated Hospital of Soochow University, Changzhou, China

**Keywords:** pancreatic cancer, PD-1, chemotherapy, immune checkpoint inhibitors, nab-paclitaxel, gemcitabine

## Abstract

**Background:**

Pancreatic cancer (PC) is widely recognized as one of the most malignant forms of cancer worldwide. Monotherapy with immune checkpoint inhibitors (ICI) has shown limited efficacy in treating this disease. There was controversy surrounding whether combining ICI with chemotherapy provided superior outcomes compared to chemotherapy alone.

**Methods:**

In this study, patients diagnosed with unresectable stage III/IV pancreatic cancer (PC) were classified as receiving programmed cell death protein 1 (PD-1) blockade plus gemcitabine and nab-paclitaxel (AG regimen) (PD-1/chemo, n=27, 50.9%) or chemotherapy alone (chemo, n=26, 49.1%) arm. The primary study endpoints included progression-free survival (PFS) and overall survival (OS), with an additional assessment of treatment-related adverse events graded as three or higher. Chi-square (χ2) statistics were employed to analyze the clinical differences between the two groups, while Kaplan-Meier curves were used to assess the difference in PFS and OS. Statistical significance was defined as *P*-values less than 0.05 (*P* < 0.05).

**Results:**

The median follow-up duration was 22 months (range 1-28 months). In the PD-1/chemo arm, the median PFS was eight months, whereas it was 3.5 months in the chemo arm (HR=0.459, 95% CI: 0.252-0.846, *P*=0.002). Furthermore, the median OS was 15 months in the PD-1/chemo arm and eight months in the chemo arm (HR=0.345, 95% CI: 0.183-0.653, *P*<0.001). Within the PD-1/chemo arm, 15 (55.6%) patients experienced grade 3 treatment-related adverse events, compared to 13 (50.0%) patients in the chemo arm.

**Conclusions:**

PD-1 blockade combined with nab-paclitaxel plus gemcitabine demonstrated superior efficacy to chemotherapy alone for unresectable stage III/IV PC patients. Future studies were warranted to identify immunosensitive patient subgroups within the PC population, ultimately leading to the development of more efficacious therapeutic strategies.

## 1 Introduction

Pancreatic cancer (PC) had the lowest 5-year relative survival rate (OS) among all malignant cancers, with only 11% (1). Pancreatic ductal adenocarcinoma (PDAC), accounting for about 90%, was the leading pathological type (2). Established treatments for unresectable locally advanced or advanced PC include the AG regimen (nab-paclitaxel in combination with gemcitabine) or the FOLFIRINOX regimen (oxaliplatin, irinotecan, fluorouracil, and leucovorin) (3). However, the survival outcomes of patients with PC treated solely with chemotherapy remained suboptimal (4). There was a pressing need for novel treatment modalities to manage locally advanced or metastatic PC that was not amenable to surgical resection.

The emergence of immune checkpoint inhibitors (ICI) therapy has significantly altered the treatment model for several cancers, including lung cancer and malignant melanoma (5). However, except for a small subset of patients (less than 1%) who exhibited microsatellite instability (MSI), ICI monotherapy has shown limited efficacy in PC (6–9). This was attributed to the immunosuppressive tumor microenvironment (TME), little immunogenicity, and low tumor mutation burden (TMB) of PC (2, 10).

Further investigation was needed to apply ICI therapy in PC, and combination therapy represented a promising avenue for exploration. Notably, there were divergent opinions regarding the superiority of ICI plus chemotherapy over standard chemotherapy in PC. Wainberg et al. (11) showed that nivolumab (PD-1 antibody) combined with nab-paclitaxel plus gemcitabine for PC was not superior to nab-paclitaxel plus gemcitabine alone in a phase I trial. Kamath et al. (12) failed to demonstrate superior efficacy of the combination of ipilimumab (CTLA-4 antibody) and gemcitabine for treating advanced PC compared to gemcitabine alone. Similarly, Fu et al. (13) also demonstrated disappointing results in the phase II clinical study, reporting no significant difference in survival benefit between sintilimab (PD-1 antibody) plus the modified FOLFIRINOX arm and modified FOLFIRINOX arm alone among 55 patients with advanced PC. However, Padrón et al. (3) demonstrated that the combination of nivolumab and gemcitabine plus nab-paclitaxel for PC resulted in a higher 1-year OS rate than gemcitabine plus nab-paclitaxel alone (57.7% vs. 35%) in a phase II trial. Gong et al. found that patients with advanced PC who received first-line ICI had longer survival (14). It was necessary to determine whether the addition of ICI to standard chemotherapy conferred superior outcomes compared to chemotherapy alone in PC.

Recently, our center has observed promising responses in patients with PC when combining PD-1 blockade with nab-paclitaxel plus gemcitabine. Therefore, we conducted a retrospective study to investigate whether PD-1 blockade combined with chemotherapy surpasses chemotherapy alone in PC. Within this retrospective study, 53 patients diagnosed with PC were enrolled to receive either PD-1 blockade combined with nab-paclitaxel plus gemcitabine or nab-paclitaxel plus gemcitabine alone. The study assesses whether PD-1 blockade combined with chemotherapy conferred superior efficacy to chemotherapy alone in unresectable stage III/IV PC patients.

## 2 Methods

### 2.1 Patients

**Figure 1:**
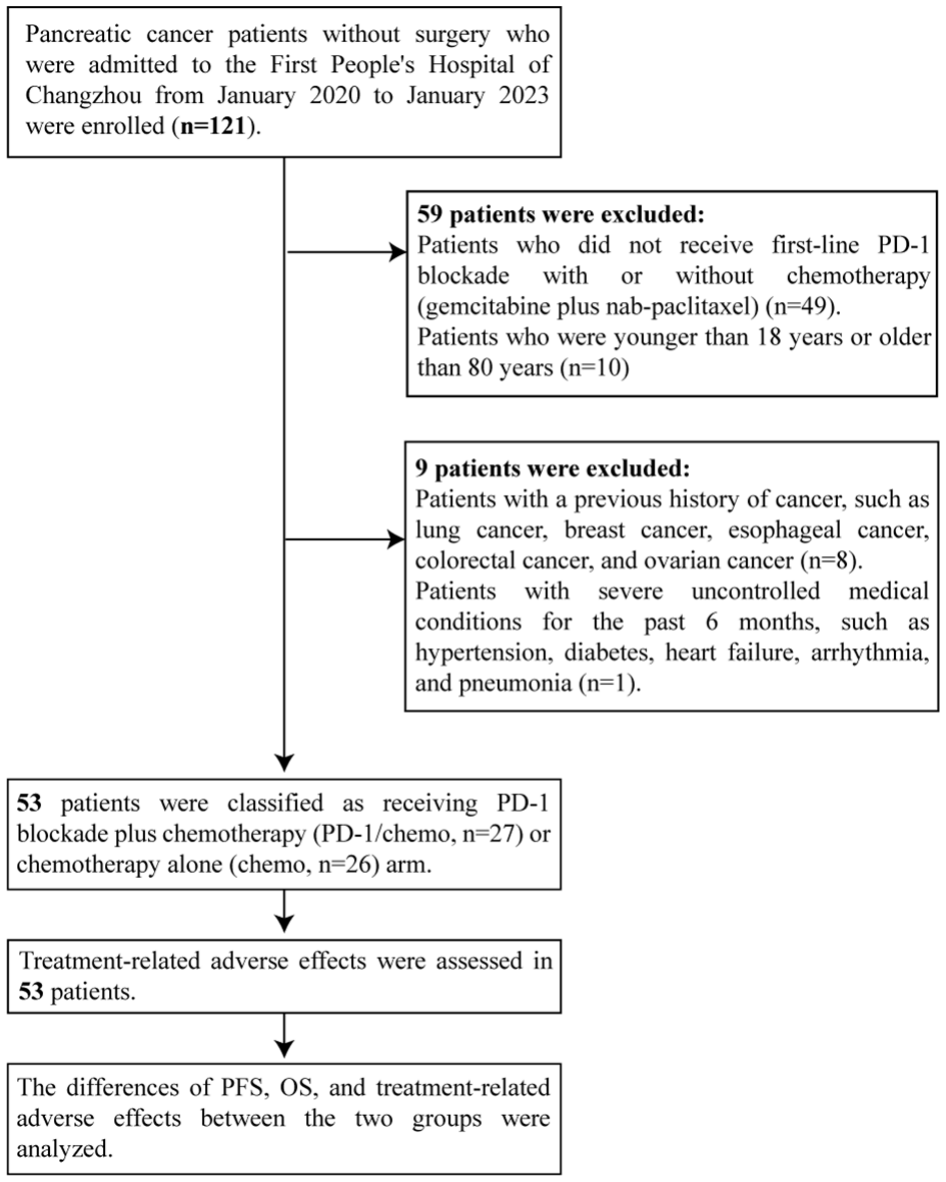
Patient flow chart. PD-1: Programmed cell death protein 1; PFS: Progression-free-survival.

From January 2020 to January 2023, 53 patients diagnosed with stage III/IV PC were enrolled at the First People’s Hospital of Changzhou. The inclusion and exclusion criteria are shown in **Figure 1**. All patients were diagnosed with primary pancreatic tumors based on histological examinations and immunohistochemical staining of fine needle aspiration biopsy specimens without undergoing surgical resection. Prior to treatment, patients underwent computed tomography (CT), magnetic resonance (MR), or positron emission tomography-CT (PET-CT) imaging, and staging was determined according to the AJCC 8th edition staging system (15). This study was approved by the Ethics Committee of the First People’s Hospital of Changzhou. Due to the retrospective nature of the study, informed consent was waived by the Ethics Committee.

### 2.2 Treatment

All PC patients received first-line PD-1 blockade with or without chemotherapy. Eight patients (29.6%) received sintilimab (200mg, day one every 21 days) in combination with gemcitabine (1000mg/m^2^, days 1 and 8 every 21 days) plus nab-paclitaxel (125mg/m^2^, days 1 and 8 every 21 days). Two patients (7.4%) received camrelizumab (200mg, day one every 21 days) combined with gemcitabine plus nab-paclitaxel. Sixteen patients (59.3%) received tislelizumab (200mg, day one every 21 days) combined with gemcitabine plus nab-paclitaxel. One patient (3.7%) received pembrolizumab (200mg, day one every 21 days) combined with gemcitabine plus nab-paclitaxel. Twenty-six patients received gemcitabine (1000mg/m^2^, days 1 and 8 every 21 days) plus nab-paclitaxel (125mg/m^2^, days 1 and 8 every 21 days).

### 2.3 Data collection and assessment

The patients with PC were classified as receiving PD-1 blockade plus chemotherapy (PD-1/chemo) or chemotherapy alone (chemo) arm. The primary endpoints of the study were progression-free survival (PFS) and overall survival (OS). The PFS was defined as the duration from treatment initiation to either disease progression or death, while OS was defined as the period from treatment initiation to death. The Response Evaluation Criteria in Solid Tumors (RECIST) version 1.1 was employed to assess disease progression (16). The Eastern Cooperative Oncology Group (ECOG) score was evaluated prior to the initiation of treatment in patients. Treatment-related adverse events of grade 3 or higher were also recorded.

### 2.4 Statistical analysis

Chi-square (χ2) statistics were utilized to evaluate the characteristic clinical differences between the two groups, while Kaplan-Meier curves were employed to assess the difference in PFS and OS. Median follow-up time was calculated using the reverse Kaplan-Meier method. Statistical significance was defined as P-values less than 0.05 (*P* < 0.05). The statistical software SPSS 25.0 and GraphPad were used for data processing.

## 3 Results

### 3.1 Baseline characteristics

Fifty-three patients were enrolled in this study, including 30 males (56.6%) and 23 females (43.4%). The median age of the study patients was 67 years, ranging from 45 to 79 years. All 53 patients (100%) were diagnosed with primary pancreas tumors. TNM stages III and IV distribution among patients with PC before treatment was 45.3% and 54.7%, respectively. The characteristics of the patients were summarized and presented in **Table 1**.

**Table 1:** Clinical characteristics.

Characteristic	Category	All Patients (n=53)	PD-1/chemo (n=27)	chemo (n=26)	*P*
Age (years)	Median (range)	67 (45-79)	64 (55-79)	68 (45-78)	
sex					0.691
	Male	30 (56.6%)	16 (53.3%)	14 (46.7%)	
	Female	23 (43.4%)	11 (47.8%)	12 (52.2%)	
Histology					0.465
Adenocarcinoma		41 (77.4%)	22 (53.7%)	19 (46.3%)	
Unknown		12 (22.6%)	5 (41.7%)	7 (58.3%)	
c-TNM					0.328
	III	24 (45.3%)	14 (58.3%)	10 (41.7%)	
	IV	29 (54.7%)	13 (44.8%)	16 (55.2%)	
Metastic site					
	Liver	25 (47.2%)	10 (40%)	15 (60%)	
	Lung	1 (1.9%)	0 (0.0%)	1 (100%)	
	Bone	2 (3.8%)	0 (0.0%)	2 (100%)	
	Lymph	18 (34.0%)	8 (44.4%)	10 (55.6%)	
	Adrenal glands	3 (5.7%)	2 (66.7%)	1 (33.3%)	
ECOG score					0.497
	0	18 (34.0%)	8 (44.4%)	10 (55.6%)	
	1	35 (66.0%)	19 (54.3%)	16 (45.7%)	
Type of anti-PD-1					
	Sintilimab	8 (29.6%)	8 (100%)	0 (0.0%)	
	Camrelizumab	2 ((7.4%)	2 (100%)	0 (0.0%)	
	Tislelizumab	16 (59.3%)	16 (100%)	0 (0.0%)	
	Pembrolizumab	1 (3.7%)	1 (100%)	0 (0.0%)	

PD-1/chemo, PD-1 blockade plus chemotherapy; chemo, chemotherapy alone; ECOG, Eastern Cooperative Oncology Group.

### 3.2 PFS and OS

**Figure 2:**
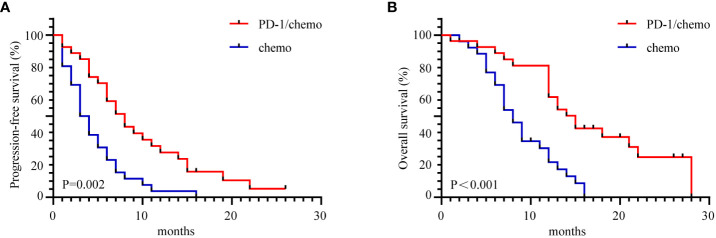
The Kaplan-Meier curves demonstrated a significant difference in PFS and OS between the PD-1/chemo arm and the chemo arm. **(A)** PFS of the PD-1/chemo arm was significantly superior to that of the chemo arm. **(B)** OS was significantly better in the PD-1/chemo arm compared to the chemo arm. PFS, Progression-free-survival; OS, overall survival; PD-1/chemo, PD-1 blockade plus chemotherapy; Chemo, Chemotherapy.

**Figure 3:**
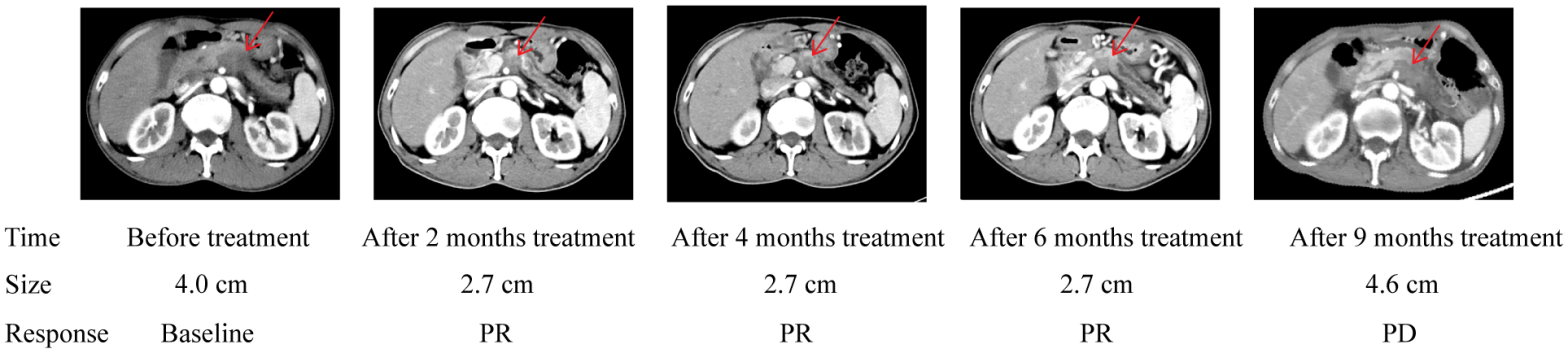
Abdomen CT of the pancreatic cancer patient treated with PD-1/chemo showed a lesion in the pancreatic. The table below shows the response evaluation results according to RECIST 1.1 criteria. PR, Partial response; PD, Progressive disease.

The median follow-up duration was 22 months (range 1-28 months). In the PD-1/chemo arm, the median PFS was eight months, whereas it was 3.5 months in the chemo arm (HR=0.459, 95% CI: 0.252-0.846, *P*=0.002). Furthermore, the median OS was 15 months in the PD-1/chemo arm and 8 months in the chemo arm (HR=0.345, 95% CI: 0.183-0.653, *P*<0.001) (**Figure 2**). Additionally, **Figure 3** showed the CT follow-up outcomes of an individual patient in the PD-1/chemo arm.

### 3.3 Safety

Within the PD-1/chemo group, 15 patients (55.6%) experienced grade 3 treatment-related adverse events including leukopenia (n=6, 22.2%), neutropenia (n=5, 18.5%), anemia (n=3, 11.1%), thrombocytopenia (n=3, 11.1%), liver dysfunction (n=2, 7.4%), immune-related pneumonia (n=2, 7.4%), and immune-related enteritis (n=1, 3.7%). In the chemo group, 13 patients (50.0%) encountering grade 3 treatment-related adverse events, encompassing leukopenia (n=5, 19.2%), neutropenia (n=6, 23.1%), anemia (n=3, 11.5%), thrombocytopenia (n=5, 19.2%), and liver dysfunction (n=1, 3.8%). Treatment-related adverse events are detailed in **Table 2**.

**Table 2:** Treatment-related adverse events.

	Grade, NO. (%)					
	PD-1/chemo arm (n=27)			chemo arm (n=26)		
	Any	1-2	3	Any	1-2	3
TRAEs	22 (81.5)	21 (77.8)	15 (55.6)	21 (80.8)	19 (73.1)	13 (50.0)
Leukopenia	14 (51.9)	8 (29.6)	6 (22.2)	14 (53.8)	9 (34.6)	5 (19.2)
Neutropenia	12 (44.4)	7 (25.9)	5 (18.5)	14 (53.8)	8 (30.8)	6 (23.1)
Anemia	11 (40.7)	8 (29.6)	3 (11.1)	8 (30.8)	5 (19.2)	3 (11.5)
Thrombocytopenia	9 (33.3)	6 (22.2)	3 (11.1)	11 (42.3)	6 (23.1)	5 (19.2)
Liver dysfunction	12 (44.4)	10 (37.0)	2 (7.4)	10 (38.5)	9 (34.6)	1 (3.8)
Kidney dysfunction	3 (11.1)	3 (11.1)	0 (0.0)	1 (3.8)	1 (3.8)	0 (0.0)
Vomit	10 (37.0)	10 (37.0)	0 (0.0)	9 (34.6)	9 (34.6)	0 (0.0)
Immune-related pneumonia	6 (22.2)	4 (14.8)	2 (7.4)	0 (0.0)	0 (0.0)	0 (0.0)
Immune-related rash	6 (22.2)	6 (22.2)	0 (0.0)	0 (0.0)	0 (0.0)	0 (0.0)
Immune-related enteritis	2 (7.4)	1 (3.7)	1 (3.7)	0 (0.0)	0 (0.0)	0 (0.0)

TRAEs, Treatment-related adverse events; PD-1/chemo, programmed cell death protein 1 (PD-1) blockade plus chemotherapy; Chemo, chemotherapy.

## 4 Discussion

In this retrospective study, the PD-1/chemotherapy arm demonstrated a notable extension in median progression-free survival (PFS) by 4.5 months when compared to the chemotherapy-only arm. Additionally, both arms exhibited similar safety profiles. Notably, the PD-1/chemotherapy combination exhibited a substantial overall survival (OS) benefit, with a 7-month improvement over the chemotherapy-only arm. This suggested that the combination of ICI and chemotherapy might offer superior outcomes compared to the current standard treatment approach of chemotherapy alone for patients with unresectable locally advanced or advanced PC.

The efficacy of ICI monotherapy in PC patients has shown limitations (17, 18), and combination treatment options are being explored. ICI in tandem with chemotherapy has exhibited reliable efficacy in various solid tumors, including lung and breast cancer (19, 20). The superiority of ICI plus chemotherapy over standard chemotherapy for PC remained uncertain. A previous study showed that the median PFS and OS of PC patients treated with gemcitabine plus nab-paclitaxel were 5.5 months and 8.5 months, respectively (21). In a phase I study by Wainberg et al. involving 50 advanced PC patients, the combination of nivolumab with nab-paclitaxel and gemcitabine yielded median PFS and OS were 5.5 and 9.9 months, respectively (11). Therefore, Wainberg et al. (11) concluded that the PD-1 blockade plus chemotherapy was not superior to chemotherapy alone for treating PC. This disparity with our research findings could be attributed to distinct usage of antineoplastic drugs, including dosage and timing. Furthermore, patient heterogeneity may also account for the conflicting results. Importantly, consistent with this study, Padron et al. (3) demonstrated that combining PD-1 blockade with chemotherapy had higher 1-year OS than chemotherapy alone (57.7% vs 35%). And in a phase Ib/II trial of pembrolizumab plus chemotherapy, 12 patients with advanced PC had a median PFS of 9.1 months and OS of 15 months (22). These findings lend further support to the credibility of our research. Based on the available evidence, future studies are needed to identify PC populations susceptible to PD-1 blockade.

Multiple mechanisms contribute to immune resistance in PC (23, 24), and this study provides evidence that nab-paclitaxel and gemcitabine can enhance the immune response in patients with PC. The underlying mechanisms warrant further discussion. The resistance of PC to ICI therapy is mainly attributed to its special TME. ICI therapy relies on immune cells, and the low mutation burden of PC leads to the lack of infiltration of active immune cells in the TME (25). On the other hand, the components of the TME in PC, including tumor-associated macrophages, myeloid-derived suppressor cells, regulatory T cells, and tumor-associated fibroblasts, collectively contribute to immunosuppression (26). Finally, the TME of PC possesses a dense connective tissue stroma (27), which results in low T cell infiltration in the TME (28–30). Nab-paclitaxel or gemcitabine may improve the response of PC to ICI therapy by acting on these resistance pathways. Von Hoff et al. (31) showed that nab-paclitaxel alone or combined with gemcitabine reduced the proliferation of connective tissue stroma in PC. This may increase the infiltration of active immune cells in the TME of PC. However, Wainberg et al. (11) did not observe an increase in the infiltration of CD8^+^ T cells and CD4^+^ T cells in the TME of PC treated with nivolumab combined with nab-paclitaxel plus gemcitabine. Given the limited number of samples (11), further investigations are warranted to determine whether paclitaxel or gemcitabine can augment immune cell activation within the TME of PC. On the other hand, the low immunogenicity of PC contributed to the resistance of ICI (17), while chemotherapy had the potential to enhance its immunogenicity (32–34). Chemotherapy could enhance ICI efficacy through its ability to increase the release of tumor antigens (35). Further research was necessary to elucidate the mechanism underlying our findings.

## 5 Limitations and prospects

The limitations inherent in this retrospective study must be acknowledged. Immune biomarkers, such as PD-L1, TMB, and MSI, were not assessed in participants prior to treatment. This limitation impeded our ability to identify a subset of patients with PC who may respond well to PD-1 blockade. The participants in this study were exclusively Chinese, potentially limiting generalizability to other populations.

Immunotherapy for PC remains a critical area of ongoing research with substantial challenges. According to the findings of our study, the combination therapy of PD-1 blockade with nab-paclitaxel and gemcitabine demonstrates the potential for enhancing clinical efficacy in a specific subgroup of patients diagnosed with PC. Future research direction lies in developing novel drug combinations aimed at enhancing the immune response against PC. For example, Rojas et al. (36) have successfully developed a personalized RNA neoantigen vaccine demonstrating the capacity to activate T cells in individuals with PC. Burrack et al. (37) demonstrated that the combination of PD-1 and PD-L1 blockade effectively reinvigorated T cells, resulting in a significant extension of survival in a murine model of PC. This drug combination presents a promising avenue for potential clinical trials. Furthermore, ongoing clinical trials exploring the combination of TIGIT/PD-1 co-blockade and CD40 agonism have exhibited substantial antitumor response in a murine PC model (38).

## 6 Conclusions

PD-1 blockade combined with nab-paclitaxel plus gemcitabine demonstrated superior efficacy to chemotherapy alone for unresectable stage III/IV PC patients. Subsequent studies should target identification of immunosensitive PC patient subgroups, paving the way for the development of more effective therapeutic strategies.

## Data availability statement

The original contributions presented in the study are included in the article/supplementary material. Further inquiries can be directed to the corresponding authors.

## Ethics statement

The studies involving humans were approved by Ethics Committee of the First People’s Hospital of Changzhou. The studies were conducted in accordance with the local legislation and institutional requirements. Informed consent was waived by Ethics Committee of the First People’s Hospital of Changzhou due to retrospective nature of study.

## Author contributions

DC: Conceptualization, Formal Analysis, Software, Writing – original draft, Writing – review & editing. JH: Conceptualization, Formal Analysis, Software, Writing – original draft, Writing – review & editing. XW: Conceptualization, Formal Analysis, Software, Writing – original draft, Writing – review & editing. BW: Supervision, Validation, Writing – review & editing. RC: Supervision, Validation, Writing – review & editing. WZ: Supervision, Validation, Writing – review & editing. CF: Funding acquisition, Supervision, Validation, Writing – review & editing. MJ: Funding acquisition, Supervision, Validation, Writing – review & editing.
